# Genes Related to Fatty Acid β-Oxidation Play a Role in the Functional Decline of the *Drosophila* Brain with Age

**DOI:** 10.1371/journal.pone.0161143

**Published:** 2016-08-12

**Authors:** António Laranjeira, Joachim Schulz, Carlos G. Dotti

**Affiliations:** 1 Center for Human Genetics, VIB—Center for the Biology of Disease, University of Leuven, 3000, Leuven, Belgium; 2 Centro Biologia Molecular “Severo Ochoa”, CSIC-UAM, Campus Universidad Autonoma de Madrid, 28049, Madrid, Spain; Biomedical Sciences Research Center Alexander Fleming, GREECE

## Abstract

In living organisms, ageing is widely considered to be the result of a multifaceted process consisting of the progressive accumulation of damage over time, having implications both in terms of function and survival. The study of ageing presents several challenges, from the different mechanisms implicated to the great diversity of systems affected over time. In the current study, we set out to identify genes involved in the functional decline of the brain with age and study its relevance in a tissue dependent manner using *Drosophila melanogaster* as a model system. Here we report the age-dependent upregulation of genes involved in the metabolic process of fatty acid β-oxidation in the nervous tissue of female wild-type flies. Downregulation of CG10814, *dHNF4* and lipid mobilizing genes *bmm* and *dAkh* rescues the functional decline of the brain with age, both at the cellular and behaviour level, while over-expression worsens performance. Our data proposes the occurrence of a metabolic alteration in the fly brain with age, whereby the process of β-oxidation of fatty acids experiences a genetic gain-of-function. This event proved to be one of the main causes contributing to the functional decline of the brain with age.

## Introduction

In nature, ageing of living organisms constitutes a complex and multilayered process characterized by the progressive accumulation of damage and a time-dependent functional decline of biological systems [1, 2]. These detrimental physiological changes are observed across multiple tissues and organs. Many of the described changes occurring with age affect genome stability [3], mitochondrial bioenergetics [4,5] and proteostasis [6] to name only a few. Whether such molecular and cellular “hallmarks” [2] constitute a direct cause or an effect of ageing remains to be fully elucidated, and the answer to this question might be highly dependent on the age, sex and environmental factors affecting a particular tissue or organism.

Among the varied approaches to the study of ageing, it stands out that many researchers have focused on the genetic manipulation of longevity, namely by identifying mutants that have prolonged/shortened life span [7, 8]. Despite being one of the more readily studied parameters of ageing, longevity in itself might constitute only a limited and general outcome of ageing, as opposed to a more centred systems function approach, which may provide more information about the interface between systems as they age. In humans, one of the most striking age-related functional deficits occurs at the level of the nervous system, reflected in a progressive cognitive decline and an increased susceptibility for the appearance of neurodegenerative diseases [9]. In parallel with humans, ageing *Drosophila* also exhibit deficits associated to a loss of neuronal function, including a progressive decline in locomotor ability and olfactory response, impairment of learning and memory and perturbations in circadian rhythm behaviour [10].

Several of the genetic modifications that successfully lead to an increase of lifespan concern genes related to mechanisms that underlie the control of basic metabolic functions, many of which are conserved across species [11]. Supporting this, we find that lowered insulin/IGF (IIS) signalling has extended lifespan in *C*. *elegans* [12], *D*. *melanogaster* [13] and *M*. *musculus* [14, 15]. This pathway is responsible for nutrient sensing and controlling glucose metabolism in many organisms. In *Drosophila*, it has been shown that loss of *chico*, the single fly insulin receptor substrate, increases lifespan together with improved immune function [16]. Also, tissue specific interventions, as brain-specific knockout of the IRS-2 in mice [17] and overexpression of dPTEN or dFOXO in the fat body of flies can extend lifespan [18, 19], both of which are downstream effectors of the IIS pathway. Furthermore, muscle-specific overexpression of dFOXO/4E-BP signalling preserves muscle function and extends lifespan in flies [20]. The pleiotropic variation of effects observed when manipulating IIS pathway enhances the importance concerning genetic manipulations of the same pathway in a different tissue / developmental stage, which can be determinant in the development or rescue of an ageing-phenotype.

The study of these evolutionarily conserved genes and pathways in a life-span context has increased the relevance of metabolic control in the development of the ageing phenotype. Despite this, the relationship between metabolic regulation and systems function in ageing remains poorly understood, especially when considering the brain.

In the current study, we set out to identify genes involved in the functional decline of the *Drosophila* brain during ageing. Here, we report that upregulation of fatty acid β-oxidation genes in the fly brain is an important determinant contributing to the neuronal functional decline that characterizes ageing female *Drosophila*.

## Material and Methods

### Fly maintenance and Collections

*Drosophila melanogaster* were cultured and kept at 25°C using standard method. Progeny was collected 24hr from eclosion and reared at standard density (25–40 flies per vial) on cornmeal/yeast fly food at 25°C (65% humidity) under a 12hr on-off light cycle. Flies were transferred into fresh food vials every other day and kept as a mixed-gender population.

For experiments using the driver *ELAV-GS-GAL4*, activation of GAL4 was achieved by transferring adult flies (1 week old) to food containing 200μM RU486 (Mifepristone: Sigma-Aldrich) which was dissolved in ethanol. Flies were maintained at 25°C, being transferred into fresh food with every other day. Flies reared on food containing equivalent amount of ethanol were used as controls.

### Fly stocks

Selected fly lines from the Genome-wide UAS-RNA interference libraries (Vienna *Drosophila* Research Center, TRiP Harvard collection and National Institute of Genetics, Japan) were used to silence ageing candidate genes. 260 genes were used in the primary screen. Tissue-specificity of the knockdown was achieved by using the transgenic GAL4-UAS system [21], a binary system composed of a driver line expressing the yeast transcription factor GAL4 cell-autonomously in the cells or tissue of interest, together with the UAS-line that drives the expression of double stranded RNA for induction of RNA interference under control of GAL4. For all experiments, *nSyb-GAL4* females were mated with male transgenic and syngenic control flies, and the resulting female offspring analyzed in parallel by comparing transgene expressing flies with age-matched control flies having the same genetic background.

Concerning the genome-wide association study we used 40 fly lines from the *Drosophila* Genetic Reference Panel (DGRP) to assay NGT phenotype. The DGRP was created by 20 generations of full sib mating progeny of wild-caught, gravid females from Raleigh, North Carolina [22].

### GWAS screen

The GWS data was obtained by calculating a pearson coefficient for the correlation of the expression of each gene and the geotaxis performance (3w vs 1w, n = 8) in the isogenic lines.

### Negative Geotaxis Assay

Negative geotaxis assay was performed based on a modified version of a previously described method [23]. Briefly, age-matched genotypes are collected under brief CO_2_ anesthesia (1-2min) and allowed to recover at least 18h-24h at 25°C prior to assay. Each NGT tube (30 cm length, 1.6cm Ø) was loaded with 10 flies of corresponding genotype. The tubes are then placed in front of a background, ready to be tested and recorded with digital camera. After initial standard trial, NGT activity was recorded by taking 3 consecutive digital images 15 sec after flies were tapped to the bottom of tubes, with a 30s rest period between recordings. Average distance of 10 flies from 3 consecutive recordings is taken into account for the calculation of average speed per genotype/vial. Genotype of interest is always tested in parallel with age-matched control. Multiple biological replicates are tested separately and taken into account for the final average speed value of performance per genotype/age. The performance was compared and quantified by image analysis using Cell B software, Olympus.

### Circadian Rhythm Assay

Circadian rhythm and activity assay was done as previously described [24]. In short, age-matched individual flies were briefly anesthetized and transferred to monitor tubes. A period of at least 24h was reserved for fly recovery and acclimatization. Behavioural activity of flies was monitored by using the infrared *Drosophila* Activity monitoring system (Trikinetics, Waltham, MA) at the standard conditions of 25°C under a dark and light cycle of 12h. The individual recordings of monitored flies over period of 24h are taking into account and averaged per monitor. Multiple monitors are used per recording session (normally 3) and multiple recording sessions are performed (with different biological samples/flies).

### Western Blot and Biochemical Analysis

Biochemical analysis of detergent-insoluble fractions was done as previously described [20,25], with few modifications. In brief, fly heads were dissected from at least 20 female flies and homogenized in ice-cold PBS with 1% Triton X-100 containing protease and phosphatase inhibitors (Roche). Homogenates were centrifuged at 14000 rpm at 4°C and supernatant collected (Triton X-100 soluble fraction). The remaining pellet was washed in ice-cold PBS with 1% Triton X-100. The pellet was then resuspended in 2% SDS, 50mM Tris pH 7.4, centrifuged at 14000 rpm at 4°C, and collected supernatants (Triton X-100 insoluble fraction) were resolved on 4–20% Tris-Glycine SDS-PAGE. Protein was quantified using BCA assay (ThermoPierce). Western blots were probed with anti-ubiquitin antibodies (P4D1, Cell Signaling; 1:1000) and anti-α-Tubulin (Sigma,#T6199) or anti-Histone H3 antibodies (Cell signalling,1:1000) as loading controls. Mean integrated densities of western bands were quantitated using ImageJ software.

### Quantitative Real-Time RT-PCR

Total RNA was prepared from at least 12 dissected *Drosophila* brain plus thoracic ganglionic mass using Trizol (Invitrogen) according to manufacturer’s instructions. The RevertAid™First Strand cDNA Synthesis Kit (ThermoPierce) was used for cDNA synthesis, and quantitative real-time PCR was performed with LightCycler® 480 SYBR Green I Master (Roche). *Actin5c* or *αTub84B* was used as normalization reference. Relative quantitation of mRNA levels was calculated using the comparative CT method. For primer used see Table 1.

**Table 1:** Forward and Reverse primers used for RT-qPCR experiments.

Gene	Forward Primer	Reverse primer
*Alpha-tubulin84B* (CG1913)	GCTGTTCCACCCCGAGCAGCTGATC	GGCGAACTCCAGCTTGGACTTCTTGC
*Actin 5c (CG4027)*	AGTCCGGCCCCTCCATT	CTGATCCTCTTGCCCAGACAA
*Bmm (CG5295)*	ATTGAAACACGGGGTCCATA	GCCAGAGTAATGGTGGAGGA
*dAkh (CG1171)*	AACGAAATGCTGCTCGAGAT	GTGTGCGTGCTAGACATCGT
*dHNF4 (CG9310)*	CAAAGGATTCTTCAGGAGGAGT	GTCCTTGTCCACAACGC
*CG10814*	TTCACAATATCTGGAGGGCAC	GAATGCTGTGGTTGATGCG

### Quantification of food intake

Quantification of food intake by female flies was performed as described in protocols previously published, by quantifying the uptake of a blue dye added to food [26, 27]. In this work, groups of five 7-day-old flies were previously selected according to genotype and transferred onto fresh food medium (formula 4–24, Carolina Biological Supply Co., Burlington, NC, USA) containing 2.5% (w/v) blue food dye (F D & C Blue Dye nr. 1). After a 30 min exposure to the labeled food, flies were anesthetized into eppendorf tubes and snap frozen in liquid nitrogen. Flies were homogenised in 200 μl if Milli Q water. Pestles used for homogenization were removed and rinsed with an additional 800 μl of Milli Q water. The resulting solution was passed through a 0.20 μm Millex filter (Millipore Corporation, Bedford) to remove debris. The samples obtained were than measured at 625nm (Ultrospec 2100 pro Spectrophotometer, Amersham Biosciences). To correct for background absorbance, reference readings were took from samples of age-matched flies exposed to non-dyed food.

### TAG measurement

Triacylglyceride quantification was similarly done as previously described [28], using as biological material 20 fly heads per condition. Protein quantification was performed using a BCA assay (ThermoPierce). Samples were assayed using a Victor machine microplate spectrophotometer at 562nm.

### Statistical Analysis

All statistical analysis was done by applying Unpaired and Paired Student’s t tests, and the analysis was performed using GraphPad Prism version 6.00 for Windows, (GraphPad Software, San Diego California USA, http://www.graphPad.com/).

## Results

### *In Vivo* Fly Genetic Screening to identify putative candidate ageing genes

**Fig 1:**
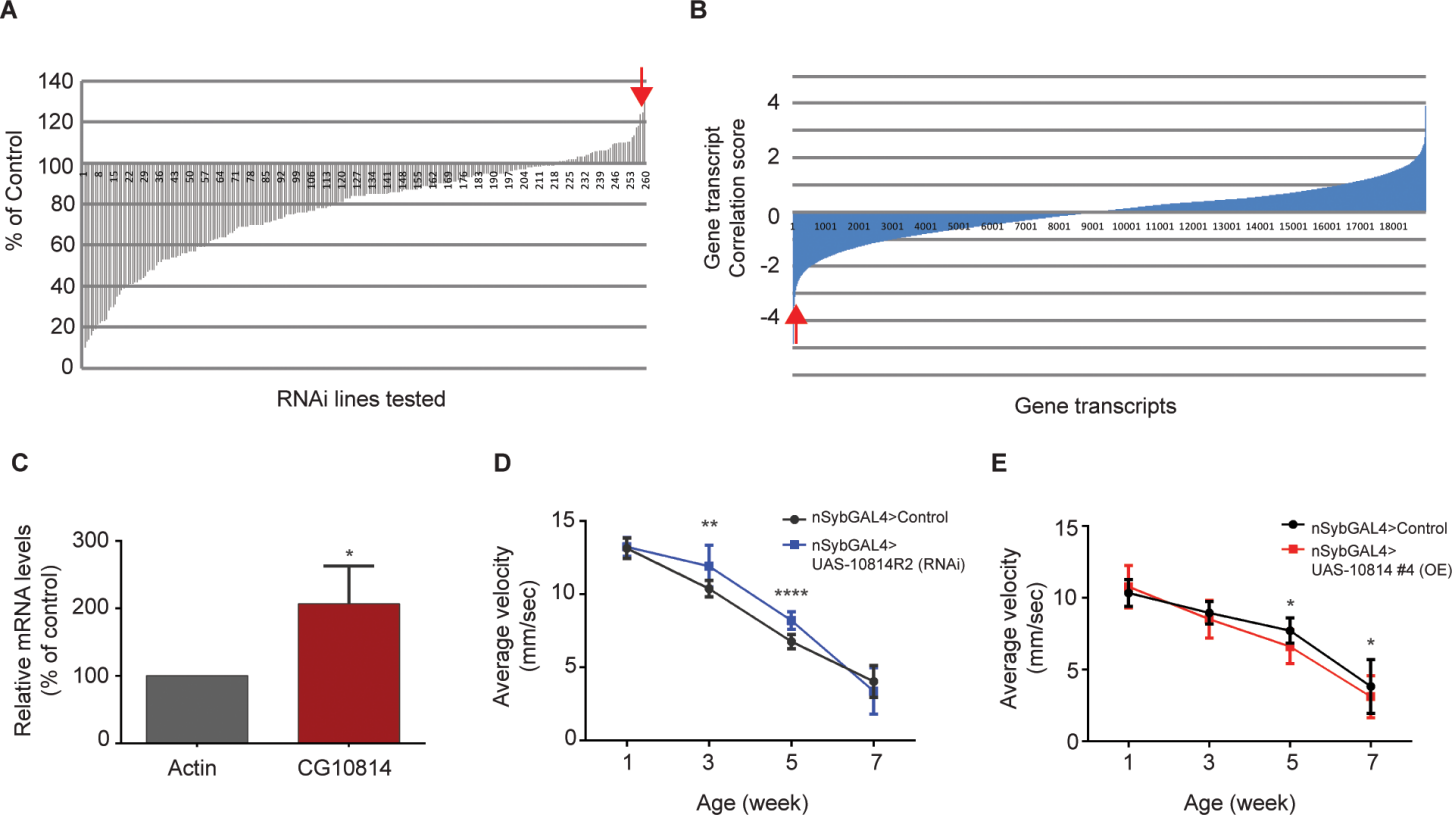
**Genetic *In vivo* screening and identification of potential genes relevant for the functional decline with age in a NGT assay**: (A), *In vivo* fly RNAi screen of approximately 260 transgenic RNAi lines resorting to neuronal specific driver *nSyb-GAL4*. Age-matched control flies and RNAi expressing flies were tested at 5W of age and their performance scored as average speed (mm/sec). (B), Genome-wide association analysis of age-dependent NGT behaviour of 40 fly lines from the *Drosophila* Genetic Reference Panel (DGRP). Fly performance was recorded at different ages and correlated with level of transcript expression in the different lines tested (Pearson correlation t-value). *Red arrow* denotes the position of CG10814, a homologue of hGBBD in both the screens performed. (C), mRNA expression levels of CG10814 from aged flies (5weeks) versus young flies (1week) (*p<0.05; SEM is indicated with n = 3; Unpaired Student’s *t*-test). (D), Negative geotaxis performance of neuronal specific knockdown of CG10814 (*nSyb-GAL4*>UAS-CG10814^RNAi^) at 1, 3, 5, and 7 weeks of age compared to age-matched control flies **p<0.01, ****p<0.0001; SEM is indicated with n = 240 flies; Paired Student’s *t*-test). Fly performance was scored as average speed (mm/sec). (E), Negative geotaxis performance of neuronal specific overexpression (OE) of CG10814 (nSybGAL4>UAS-CG10814^wt^) at 1, 3, 5, and 7 weeks of age compared to age-matched control flies. (*p<0.05; SEM is indicated with n≥210 flies; Paired Student’s *t*-test).

In order to identify potential candidate genes relevant for the functional decline of the brain with age, we performed an *In vivo* Fly RNAi Screen. As a primary screening tool, we made use of the well characterized Negative Geotaxis behaviour assay (NGT) [23]. This is a conduct known to decline with age in *Drosophila*, whereby we record the inner orienting response and movement of flies against opposing gravitational forces, elicited by a physical stimulus. In this screen, we used RNA interference (RNAi) to silence selected genes of interest in the fly brain (Fig 1A), making use of the neuronal specific driver, *nSyb-GAL4*. In total, we screened 260 transgenic RNAi lines (S1 Table), targeting genes reported to have increased genetic expression with age in *Drosophil*a [29]. The potential functional rescue of these genes’ silencing was assessed against the age-dependent loss of performance seen in wild-type background flies in the NGT assay at 35 days (5 weeks). RNAi silencing of most of the genes resulted in a worsening of performance at the selected time when compared to control (Fig 1A). However, the knockdown of a small group of genes led to a rescue of performance. Among these featured CG10814 gene (Fig 1A, red arrow), a putative orthologue for the human Gamma-Butyrobetaine dioxygenase (hGBBD), a protein responsible for the last step in Carnitine biosynthesis. Carnitine is the primary metabolite involved in the transport of activated long-chain fatty acids from the cytosol to the mitochondria [30], where β-oxidation occurs.

In parallel to this effort, we complemented our initial RNAi screening approach with another analysis by performing a genome-wide association study resorting to the readily available fly genetic library–the *Drosophila melanogaster* Genetic Reference Panel [22] (*Dm*GRP). By making use of a collection of fully sequenced inbred lines derived from a single outbred population, correlations between molecular genetic variation in these lines and variation in quantitative traits can be accessed and studied, thus assigning a higher genetic relevance to the analysis of different variants in a controlled genome-wide context. To this end, we tested 40 lines of this collection at different ages in our age-dependent assay of NGT, for subsequent correlation between the different known transcript expression levels in these flies and their behaviour performance in the NGT test (Fig 1B). Again CG10814 emerged as having a potential role in ageing, by being one of the transcripts with the highest negative correlation identified (Fig 1B, red arrow), in this particular instance: i.e. higher gene expression of CG10814 was highly correlated with a decrease in performance, indicating that among the fly lines tested, an inherent increase in the levels of transcript of CG10814 in different lines was associated with a decrease in performance in the NGT assay. This further ascertained the importance of CG10814 in the development of the ageing phenotype in a controlled and natural genetic background.

### RNAi silencing of CG10814 rescues the age-dependent NGT loss of function

In humans, GBBD is expressed in kidney, liver and to a smaller extent in the brain, with different patterns of expression occurring across species [30]. In the case of *Drosophila*, based on protein sequence analysis, four homologues of *hGBBD* are found. Among these, CG10814 is one of the most likely candidates to encode the putative *dGBBD*, since like the *hGBBD* gene, CG10814 does not possess a mitochondrial targeting sequence. To confirm the potential gain-of-function with age of CG10814 at the transcriptional level, we measured changes in the transcript expression of CG10814 in young versus old fly brains (5 vs. 1 week) (Fig 1C). We observed that CG10814 transcription levels suffered a drastic increase in expression with age (Fig 1C), in contrast with the transcripts levels of other possible homologue genes related to hGBBD and the carnitine biosynthesis pathway (S1 Fig).

To validate whether high levels of CG10814 play a role in the age-dependent brain function decay in *Drosophila*, we knocked down its expression in the nervous tissue (*nSyb-GAL4*>UAS-CG10814^RNAi^, S2 Fig) and tested these flies in the NGT assay at different ages (Fig 1D). Neuronal CG10814 knockdown female flies showed enhanced performance when compared to age-matched controls in an age-dependent manner, namely at 3W and 5W (Fig 1D) (and 5W and 7W with an alternative line, S3 Fig). It should be noted that no differences were detected at a young age (1W), in either of the knock down lines tested (*nSyb-GAL4*>UAS-CG10814^RNAi^ and *nSyb-GAL4*>UAS-TRiPCG10814^RNAi^) when compared to age-matched controls, thus excluding possible developmental effects.

To further determine that increased levels of CG10814 have a negative impact in the brain ageing phenotype, we tested flies overexpressing CG10814 specifically in the neurons (*nSyb-GAL4*>UAS-CG10814^wt^) in the NGT assay at different ages (Fig 1E). We observed a significant decrease in performance of overexpressing female flies, namely at 5W and 7W of age that reflects a lower average speed of these flies when compared to control (Fig 1E).

Dietary restriction (DR) has been shown to induce changes in activity levels and have a striking impact on the life-span of flies [26, 31]. Furthermore, fly populations kept under caloric restriction have higher activity levels than populations kept on a high-caloric diet [26]. In view of these results, we hypothesized if the rescue effects observed in CG10814 RNAi flies were due to a change in the feeding behavior that could ultimately result in a DR-like condition. In order to verify this possibility, we measured the fly food intake by tracking the amount of label (F D & C Blue Dye nr. 1) ingested by the CG10814 RNAi flies and age-matched controls (S4 Fig). We observed that knockdown of CG10814 in the nervous system of flies induced no significant changes in the amount of food label ingested when compared to age-matched control (S4 Fig), therefore excluding any possible DR effect derived from the genetic downregulation of CG10814.

Together, these data support the idea that age-associated increase of CG10814 expression in the *Drosophila* brain produces a gain-of-function effect, adversely affecting normal neuronal function in NGT assay.

### RNAi silencing of CG10814 rescues ageing circadian rhythm parameters in old flies

To complement the results observed in the NGT assay, we performed an analysis of the age-associated changes in sleep:wake cycles in aged flies. Loss of sleep consolidation with age is a common feature in many ageing organisms, including fly, mouse and human, which share molecular and functional similarities in respect to this behaviour [24]. Flies exhibit a pattern of sleep fragmentation in old age that results in increased daytime sleep and increased night time wakefulness, reminiscent of that seen in humans [24, 32].

**Fig 2:**
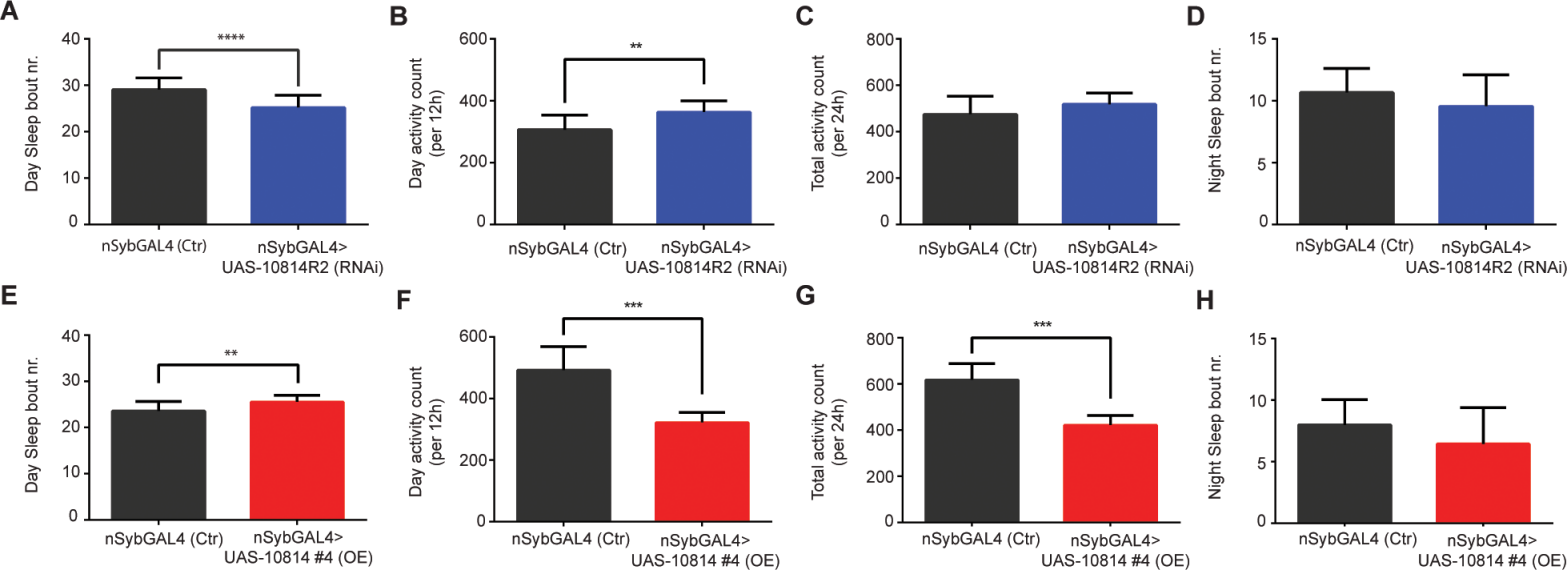
**CG10814 knockdown in the nervous tissue results in a rescue of age-dependent loss of sleep consolidation**: Comparison of circadian/activity parameters of neuronal specific knockdown of CG10814 (nSybGAL4>UAS-CG10814^RNAi^) flies with age-matched controls, at 5 weeks of age—(A), Day Sleep bout number; (B), Day activity counts (per 12h period); (C), Total activity count (per 24h period); (D), Night Sleep bout number (**p<0.01, ****p<0.0001; SEM is indicated with n = 192 flies; Paired Student’s *t*-test). Comparison of circadian/activity parameters of neuronal specific overexpression (OE) of CG10814 (*nSybGAL4*>UAS-CG10814^wt^) flies with age-matched controls, at 5 weeks of age. (E), Day Sleep bout number; (F), Day activity counts (per 12h period); (G), Total activity count (per 24h period); (H), Night Sleep bout number (**p<0.01, ***p<0.001; SEM is indicated with n = 144 flies; Paired Student’s *t*-test).

To ask whether this physiological function could be rescued in old flies (5W) by downregulating CG10814, we monitored the effect of the neuronal-specific knockdown on the different ageing parameters that reflect the sleep fragmentation and breakdown of rhythm strength that characterizes old age (Fig 2). We observed that in CG10814 neuronal knockdown female flies, the number of day sleep bouts (periods) is reduced when compared to age-matched control flies (Fig 2A), in parallel with this result, the number of day activity counts is also increased (Fig 2B). On the other hand, no significant differences were found in terms of total (day+night) (Fig 2C) and night activity readings (Fig 2D). Altogether, these data indicate that sleep fragmentation is ameliorated in neuronal knockdown flies when compared to age-matched control flies especially during day time, by rescuing activity and suppressing number of sleeping periods, which have been shown to increase with age [24]. It should be noted that no major differences were detected at a young age (1W), (*nSyb-GAL4*>UAS-CG10814RNAi) when compared to age-matched controls (S5 Fig) (apart from day sleep bout nr.), thus asserting the age-dependent nature of the rescue.

We next asked whether increased levels of CG10814 would elicit a negative effect on sleep rhythm parameters of aged female flies, by inducing its upregulation in neurons (*nSyb-GAL4*>UAS-CG10814^wt^). This genetic approach resulted in an increase in day sleep bout number (Fig 2E) and a decrease in the day activity counts (Fig 2F) when compared to control flies, meaning increased number of inactive (sleep) periods during the day. In addition, we observed an overall decrease in total activity count (Fig 2G) which might be indicative of a broader impairment of systems function, shown by a loss of sleep:wake states (activity:inactivity) in detriment of a more general inactive like state.

### Knockdown of CG10814 rescues loss of Proteostasis that accompanies ageing

**Fig 3:**
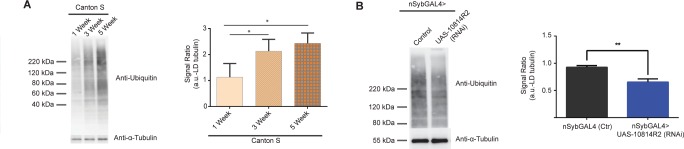
**CG10814 knockdown in the nervous tissue results in a rescue of age-dependent ubiquitination profile:** (A), Ubiquitin levels (indicative of protein aggregates) detected in Triton X-100 insoluble fraction of heads from wild type Canton S aging flies, at 1, 3, 5, and 7 weeks of age and respective quantification of ubiquitin-conjugated proteins normalized to α-tubulin levels (*p<0.05; SEM is indicated with n = 4; Paired Student’s *t*-test); (B), Ubiquitin levels corresponding to Triton X-100 insoluble fraction extracted from heads of neuronal specific knockdown of CG10814 (*nSybGAL4*>UAS-CG10814^RNAi^) flies compared to age-matched controls, at 5 weeks of age (representative of 3 blots) and D) respective quantification of ubiquitin-conjugated proteins normalized to α-tubulin levels (**p<0.01; SEM is indicated with n = 3; Paired Student’s *t*-test).

Overall progressive decline in systems function is paralleled at the cellular level by an impairment of homeostatic processes [1]. It has been shown that particular genetic interventions can rescue age-dependent functional deficits, like the maintenance of protein homeostasis [20]. Therefore, we next analysed the accumulation of misfolded proteins and the constitution of harmful protein aggregates by tracking the increase in the amount of ubiquitinated proteins with age, a common marker of cell damage and ageing [20, 29]. We observed a progressive and significant increase in the level of ubiquitination in aged wild-type flies (Fig 3A). On the other hand, downregulation of CG10814 in the nervous tissue resulted in a significant reduction in the population of ubiquitinated products (Fig 3B and S6 Fig) of 5 week old flies and thus ameliorated the loss of protein homeostasis during brain fly ageing, being indicative of a rescue at the cellular level.

To further investigate a potential underlying oxidative stress resistance associated with the rescue of function observed in the behavioural assays, we examined the effect of CG10814 knockdown on the levels of 4-HNE-protein adducts, which represents a biomarker for accumulated lipid oxidation [33,34]. We observed that CG10814 neuronal specific knockdown reduced 4-HNE-protein adducts in the heads of female flies (S7 Fig). This result suggests that CG10814 knockdown alleviates oxidative cellular damage, together with protein homeostasis.

### Genes involved in β-oxidation of fatty acids are upregulated with age

**Fig 4:**
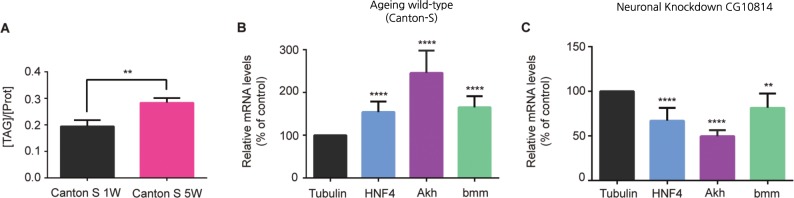
*HNF4* and lipid mobilization genes *dAkh* and *bmm* are upregulated with age in wild-type flies (Canton S) together with TAG content in fly heads. (A), Biochemical comparison of Triacylgyceride content in young and old fly heads. Amount of TAG was normalized to the amount of protein. (**p<0.01; SEM is indicated with n = 4; Paired Student’s *t*-test). (B), Relative changes in mRNA transcript levels of *HNF4*, *dAkh* and *bmm* in ageing wild-type (Canton S) fly brains (5 week vs 1 week). *αTub84B* was used as normalization reference (****p<0.0001; SEM is indicated with n≥3; Unpaired Student’s *t*-test). (C), Relative quantification of *dHNF4*, *dAkh* and b*mm* mRNA levels from brains of neuronal specific knockdown of CG10814 (*nSybGAL4*>UAS-CG10814^RNAi^) flies compared to age-matched controls, at 5 weeks of age. *αTub84B* was used as normalization reference(**p<0.01, ****p<0.0001; SEM is indicated with n≥6; Unpaired Student’s *t*-test). CG10814 loss of expression (knockdown) in fly brain down regulates expression of genes involved in activation of β-oxidation and lipid metabolism at 5W.

The predicted gene ontology function associated to CG10814 is as an enzyme involved in the biosynthesis of Carnitine, a “carrier” metabolite with an essential role in the transport of activated long-chain fatty-acids (LCFAs) from the cytosol to the mitochondria [30, 35], where β-oxidation occurs. In this context, the effects produced by the excess of CG10814 can be potentially ascribed to a role in indirectly mediating fatty acid β-oxidation in *Drosophila*. Initially, to test the relevance of this metabolic process in the brain of ageing flies, we first analyzed the triacylglyceride (TAG) content, since TAG constitutes a primary storage unit for fatty-acids, which is most commonly found in the fat body of flies. We observed that TAG levels increase with age in the head of ageing wild-type female flies (5 vs 1 week) (Fig 4A).

To further validate that increased fatty-acid metabolism plays a role in age-associated brain dysfunction, we focused our efforts on *Drosophila HNF4* (*dHNF4*), a well described gene responsible for driving fatty acid oxidation for energy production [28]. *dHNF4* has been shown to be highly expressed in tissues that control metabolism, like the midgut and fat body, with low levels being detected in the brain [28]. *dHNF4* mutants are starvation sensitive and characterized by a down regulation of genes involved in the β-oxidation pathway, and also lipid mobilizing genes under starved conditions when compared to controls [28], being unable to access TAG and long-chain fatty acid stores upon starvation.

Since *dHNF4* constitutes a general regulator of β-oxidation in the fly, we investigated possible changes occurring in the genetic expression of *dHNF4* in the brain of female wild-type flies with age. We found that transcript levels of *dHNF4* were upregulated with age showing an approximate increase of 50% between 1 and 5 weeks (Fig 4B). Similar results were also obtained when checking for the expression levels of genes involved in promoting lipid mobilization [36]–*Adipokinetic hormone* (*dAkh)* and *brummer* (*bmm*) (Fig 4B). As observed for *dHNF4*, the transcript levels of *dAkh* and *bmm* were elevated with age in neuronal tissue of wild type female flies (Fig 4B), namely 145% and 65% respectively. To determine the impact of silencing CG10814 on the brain expression levels of genes involved in β-oxidation, we checked in these flies for the transcript levels of *dHNF4*, *dAkh* and *bmm* at 5 weeks of age. We found that the neuronal knockdown of CG10814 leads to a significant decrease of 33% in the expression levels of *dHNF4* (Fig 4C). Also *dAkh* and *bmm* levels were significantly reduced through the knockdown of CG10814, 51% and 20% respectively (Fig 4C). Altogether these results demonstrate ageing is accompanied by a general gain of function of fatty acid oxidation in the brain, both at the level of TAGs and expression levels of related genes, principally *dHNF4*. It also suggests that the knockdown of CG10814 in neurons leads to a block in lipid mobilization and also a down regulation of genes essential for fatty acid β-oxidation, possibly by impeding transport of fatty-acids into the mitochondrial matrix where β-oxidation occurs.

### RNAi silencing of *dHNF4* and other lipid mobilizing genes ameliorates loss of function in aged female flies

**Fig 5:**
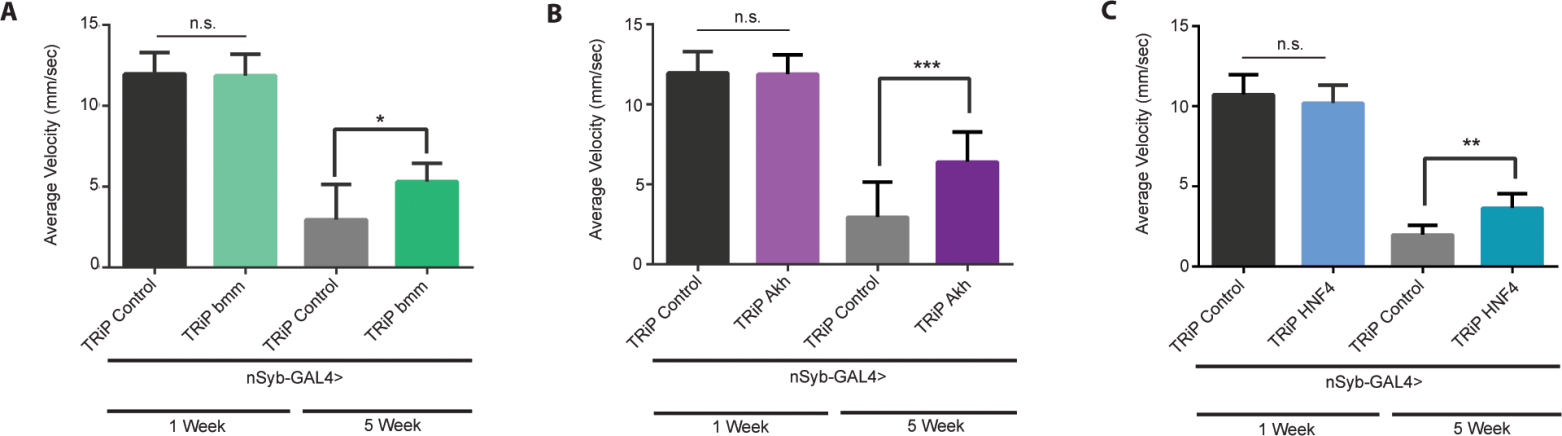
**Down regulation of fatty-acid metabolism in the Drosophila nervous tissue rescues loss of performance with age**: Negative geotaxis performance of neuronal specific knockdown of, (A), *bmm*, (B), *dAkh* and (C), *dHNF4*, at 1 and 5 weeks of age compared to age-matched control flies (respectively, *nSybGAL4*> UAS-TRiP *bmm*, UAS-Trip *Akh* and UAS-TRiP *HNF4*) (*p<0.05, **p<0.01, ***p<0.001; SEM is indicated with n = 90 flies per age group (*bmm* & *Akh*); n = 180 flies per age group (*HNF4*); Paired Student’s *t*-test).

As a proof of concept that the upregulation of genes involved in mitochondrial β-oxidation of fatty-acids have a critical role in loss of function that accompanies age, we set out to investigate the effects of the neuronal downregulation of *dHNF4*, *dAkh* and *bmm* genes. Our observations revealed that knockdown of these genes led to a significant rescue of NGT performance in aged flies at 5 weeks of age (Fig 5). It should be noted that the levels of NGT performance did not change significantly between control and knockdown lines tested at 1 week of age (Fig 5), consistent with the apparently normal developmental progression of these animals and the minor role played by these genes in the nervous system of young flies.

**Fig 6:**
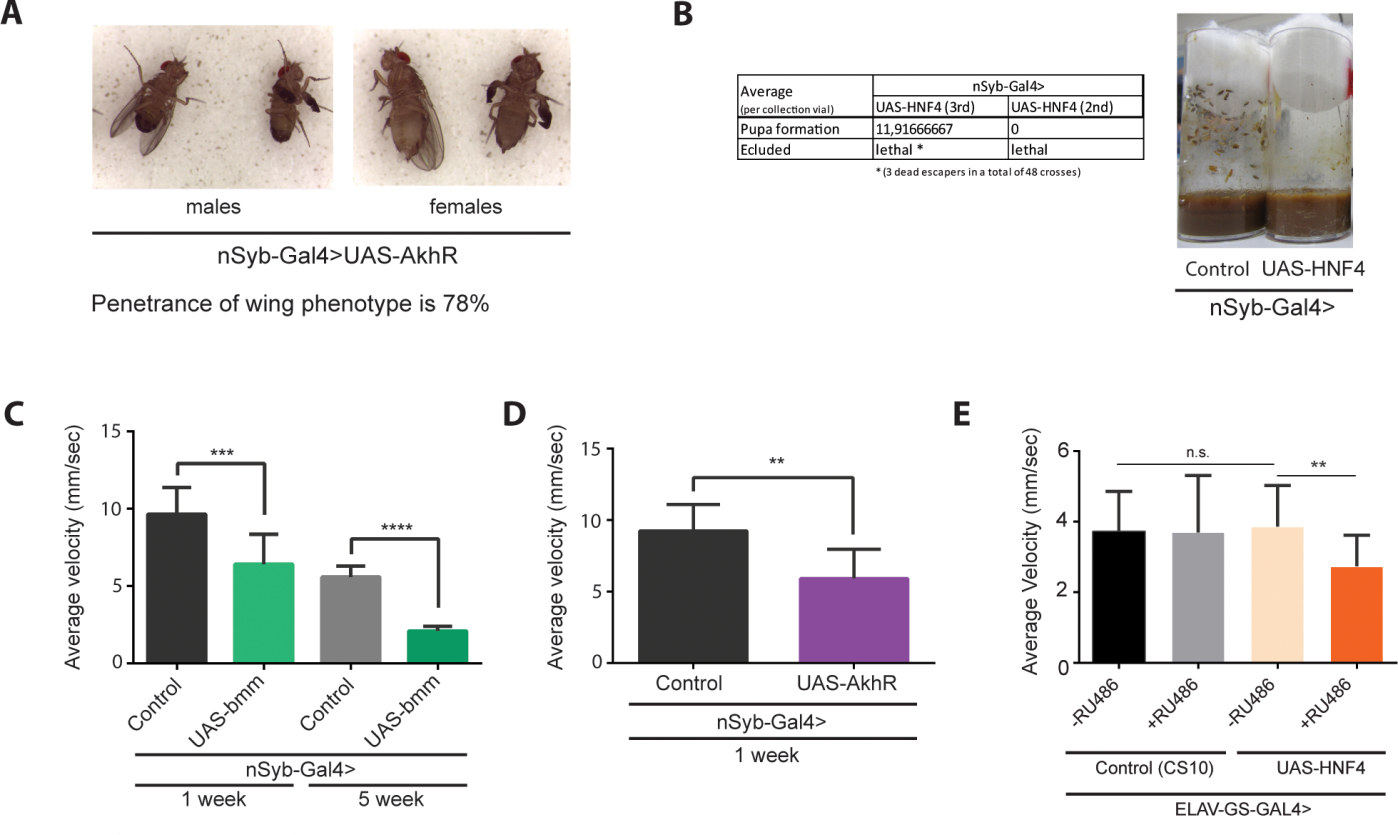
**Developmental and adult stage effects induced by neuronal overexpression of *AkhR*, *bmm* and *dHNF4*** (**A**) Description of lethality effect elicited by overexpressing *dHNF4* specifically in neuronal tissue resorting to two independent lines (on the 2^nd^ and 3^rd^ chromosomes). Picture represents the comparison of pupa formation between flies overexpressing *dHNF4* (*nSyb-GAL4>UAS-dHNF4*) and age-matched control flies. (**B**) Picture denoting wing phenotype developed through overexpression of *AkhR* in neuronal tissues. Penetrance of wing phenotype was 78%. (**C**) Negative geotaxis performance of neuronal overexpression of *AkhR*. (*nSyb-GAL4*>UAS-*AkhR*) at 1 week of age compared to age-matched control flies. (**p<0.01; SEM is indicated with n = 140 flies; Paired Student’s *t*-test) (D) Negative geotaxis performance of neuronal overexpression of *bmm* (*nSyb-GAL4*>UAS-*bmm*) at 1 and 5 weeks of age compared to age-matched control flies. (***p<0.001;****p<0.0001; SEM is indicated with n = 180 flies; Paired Student’s *t*-test). (E) Negative geotaxis performance of neuronal overexpression of *dHNF4*. Induction of GAL4 (OE) started at 1 week of age (*ELAV-GS-GAL4*>UAS-*dHNF4*) and flies were tested at 3 weeks of age (2 week treatment period with 200μM of RU486). (**p<0.01; SEM is indicated with n = 120 flies; Paired Student’s *t*-test).

To further evaluate if increased genetic expression of β-oxidation genes had a causal relationship with the loss of function observed at old age, we overexpressed *dHNF4*, *dAkhR* and *bmm* in neuronal tissue using *nSyb-GAL4* driver (Fig 6A–6D). The overexpression of such genes had a drastic detrimental effect in the development of these flies. Overexpression of *AkhR* induced a wing phenotype with a 78% penetrance (Fig 6A). *dHNF4* overexpression resulted in a severe lethal phenotype (Fig 6B) confirmed by using two alternative lines. The overexpression of *bmm* did not induce any abnormal wing or early lethal phenotype. Despite this, the overexpression of *bmm* resulted in a decrease in NGT performance both at 1W and 5W of age when compared to age-matched controls (Fig 6C), denoting its negative impact on neuronal function even at a young age (1W). Also, the overexpression of *AkhR* led to a decrease in performance in NGT assay at a young age (1W) (Fig 6D), mimicking the results observed for *bmm*. No NGT data was collected at 5W of age due to a strong life-span reduction in flies overexpressing *AkhR*.

To further determine the effect of excess *dHNF4* on adult neuronal function, we overexpressed *dHNF4* under the control of an inducible GAL4 driver (*ELAV-GS-GAL4*) specifically in neurons [37] (Fig 6E). Neuronal overexpression of *dHNF4* was induced in the young adult at 1 week of age by adding RU486 to the culture medium transgenic flies (ELAV-GS-GAL4>UAS-*dHNF4*). The presence of RU486 will activate the neuronal expressed chimeric protein *(ELAV-GS-GAL4)* containing both GAL4 DNA-binding domain and human progesterone receptor ligand-binding domain. The resulting activated chimeric molecule will bind to the UAS, ultimately driving the downstream (over)expression of *dHNF4* in neurons under the UAS control. After 2 weeks of treatment, we observed that overexpression of *dHNF4* had a negative effect on NGT performance translated into a reduced average velocity when compared to genotype/age-matched controls (Fig 6E). Also of note, RU486 treatment by itself does not affect performance in the NGT assay as seen in controls: *ELAV-GS-GAL4*>CS10 +/- RU486 (Fig 6E).

**Fig 7:**
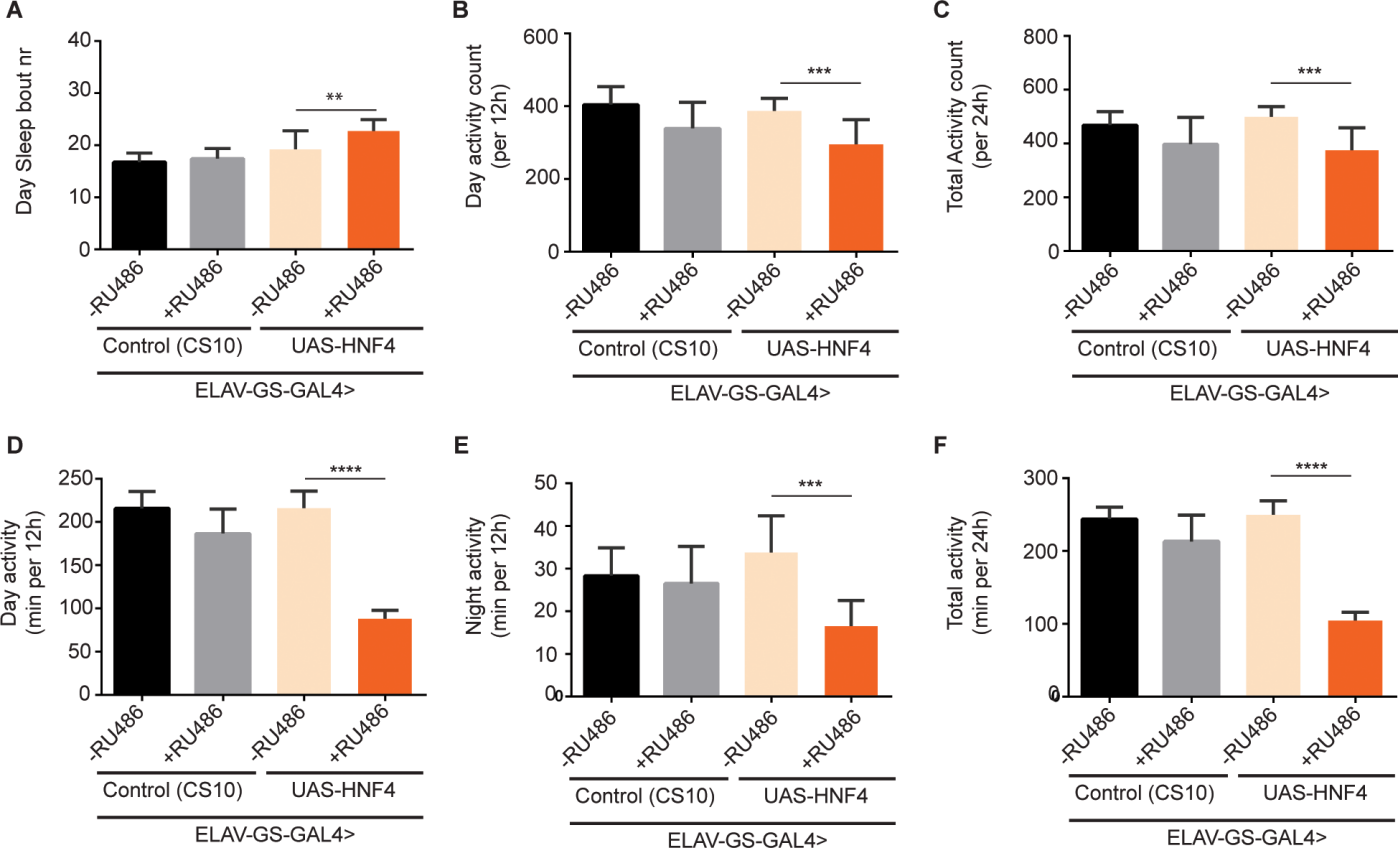
Comparison of circadian/activity parameters of neuronal specific overexpression (OE) of *dHNF4* (*ELAV-GS-GAL4*>UAS-*dHNF4*) with age-matched controls. Induction of GAL4 (OE) started at 1 week of age and flies were tested at 3 weeks of age (2 week treatment period) with 200μM of RU486. (**A**), Day Sleep bout number; (**B**), Day activity counts (per 12h period); (**C**), Total activity count (per 24h period); (**D**), Day activity (min per 12h); (**E**) Night activity (min per 12h) and (**F**) Total activity (min per24h)(**p<0.01, ***p<0.001, ****p<0.0001; SEM is indicated with n = 48 flies; Paired Student’s *t*-test).

In contrast to the dHNF4 gain-of-function, the rescue effects promoted by the *dHNF4* knockdown in the NGT performance of aged flies were further reinforced by the rescue of particular sleep consolidation parameters in old 5 week female flies, notably by decreasing the number of day Sleep bouts and increasing the night sleep bout average duration, (S8A and S8C Fig, respectively). This represents a rescue of sleep consolidation to the night/dark circadian period, by reducing sleeping periods during daytime (S8A Fig) and having longer (S8C Fig) but fewer (S8D Fig) periods of sleep during night time. In parallel to this approach, inducible overexpression of *dHNF4* also had a general detrimental effect in general activity/circadian rhythm parameters, in part by inducing an increased number of individual sleeping periods during the day (Fig 7A). More importantly, the upregulation of *dHNF4* led to a general phenotype of inactivity, whereby, either by individual counts (Fig 7B and 7C) or amount of time (Fig 7D–7F), in overall the activity parameters were reduced in comparison to controls (Fig 7B–7F), reminiscent and even more accentuated than the case observed for CG10814.

Taken together, these results highlight the pivotal role of *dHNF4* and mitochondrial β-oxidation in the development of a detrimental ageing phenotype in neuronal tissue.

## Discussion

One of the main aims of ageing research is to identify the processes that contribute to the appearance of age-related damage and loss of function in cells, tissues and organs. Crucial to this understanding is the how and where this damage occurs [1].

Increasing evidence has accentuated the fundamental role played by metabolic processes and the regulation of such in affecting specific cellular ageing events [2], which in turn affect and influence a cell’s ability to function and/or dye. At the crossroads of these metabolic regulations we find the mitochondria, the principal responsible for the metabolic homeostasis of cells. Mitochondria are considered to have a central role in the development of the ageing-phenotype and many neurodegenerative diseases [38]. With age, mitochondrial integrity and biogenesis is compromised [5], this is reflected in a reduced ability to produce ATP [4, 39], accumulation of mutations and deletions in mtDNA [40] and oxidation of mitochondrial proteins [41]. In animal cells, mitochondria are the main subcellular organelle where β-oxidation occurs, generating energy through the oxidation of long-chain fatty acids. β-oxidation consists of a repetitive four-step enzymatic process, resulting in the production of Acetyl-CoA, that can be fully oxidized in the citric acid cycle [42]. The transport of activated long-chain fatty acids into the mitochondrial matrix for β-oxidation is enabled by Carnitine, a metabolite that when transiently linked to long-chain fatty acids by transesterification permits its diffusion through mitochondrial membranes, aided by specific transporters [30, 43]. The carnitine mediated entry of long-chain fatty acids into the mitochondrial matrix is considered to be the rate limiting step for β-oxidation [30].

Our work has focused on the relevance of metabolic control in the development of the ageing phenotype, particularly in the brain. It is generally accepted that the nervous tissue engages primarily in glycolysis and oxidative phosphorylation as energy sources, as opposed to a more lipid-based metabolism through the β-oxidation of fatty-acids, a process long thought not to be as relevant for energy generation in the adult brain. Here we report that with age there is an upregulation of fatty-acid β-oxidation and lipid mobilization genes in the female fly brain, together with an increase in TAG levels and a decrease in functional performance. In parallel, we showed that genetic silencing of genes related to β-oxidation improved neuronal function at a cellular and behaviour level in old flies, namely at 5W. Due to the tissue specific nature of this effect, neuronal specific rescue of loss of performance at ages close to end of life (7W) is naturally difficult to observe since age-dependent dysfunction in other systems might have a cumulative effect on the overall functional performance of the organism. This fact might explain the variation in rescue at 7W between the two knockdown lines against CG10814.

Additionally, we demonstrated that upregulation of CG10814 and *dHNF4* in neurons aggravated the functional loss observed in old flies. The detrimental effects of such upregulation were more accentuated in the case of *dHNF4*, either during development or at adult stage. This observation can be explained due to its centre role in promoting β-oxidation and lipid mobilization through the activation of several transcriptional targets involved in the latter processes [28]. In contrast, the upregulation of CG10814 elicits a negative impact on functional performance mainly at 5W and 7W. The absence of detrimental effects at younger ages can be explained in light of its lipid-shuttling function, which is more dependent on the brain TAG accumulation with age.

Our data suggests that an increase of TAG in the brain can be of focal importance, especially when considering that it has been shown that a high-fat diet increases L-Carnitine synthesis [44] and that mammalian HNF4 isoforms [45], in parallel with dHNF4 [28], can be transcriptionally activated by ligation with long-chain fatty acids. Such activation of dHNF4 together with its role in driving a genetic program of lipid mobilization and β-oxidation could potentially lead to a feed forward process, especially in the presence of amounting TAG.

One of the possible explanations for the increased TAG content in the brain could be derived from the fact that impaired mitochondrial function observed with age contributes greatly to the increase in oxidative stress [5]. The propensity for the malfunction of the mitochondrial electron chain with age results in an increase of free radicals and Reactive Oxygen Species (ROS) production in the cellular environment. It has been demonstrated that an increase in ROS and oxidative stress in *Drosophila* neurons can lead to the accumulation of Lipid Droplets (LD) in glia [46, 47]. In parallel, it cannot be excluded that dysregulation of lipid metabolism in other tissues, like the fat body, can also contribute to this occurrence.

It should be pointed that reducing expression of lipases, such as bmm and Akh, could potentially lead to an increase in LD population. Despite this, our data shows that downregulation of bmm and Akh promotes the rescue of age-dependent decline in neuronal function (Fig 5). This further re-enforces the notion that, it is not the accumulation of lipids (TAGs) in the brain, but their mobilization and use in driving β-oxidation that is primarily responsible for the deleterious effects observed with age. Future investigations should focus on studying changes in lipid content and form (free fatty acids/TAG/Lipid Droplets) with age, and how these changes influence neuronal metabolic pathways.

It is interesting to notice that processes known to have an effect on lipid homeostasis, like calorie restriction and modulation of specific signalling pathways (e.g. downregulation of insulin/IGF pathway), are known to increase the longevity of organisms [8,48]. One of the more striking metabolic changes induced by calorie restriction is the reduction in fat tissue [48], a feature known to be sufficient in prolonging the lifespan of mice [49]. In *Drosophila*, a heterozygous mutation in *Enigma* (*Egm)*, an essential gene encoding a protein homologous to the enzymes that catalyse the first reaction of the repetitive cycle of the β-oxidation, was shown to extend lifespan and increase tolerance of flies against oxidative stress [50]. These results suggest that regulation of the β-oxidation pathway is relevant for lifespan determination and stress resistance.

In the context of these findings, our research further explores and uncovers a link between loss of neuronal function with age and regulation of β-oxidation. In the future, it would be interesting to explore and confirm how β-oxidation is regulated in different tissues and how its regulation influences the function of different systems with age. The tissue-specific nature of this work confirms that the interplay between maintenance and regulation of metabolic processes, and their occurrence and predominance in different tissues as we age, is pivotal in defining the ageing phenotype. Taken together, our data proposes that the activation and upregulation of mitochondrial β-oxidation genes plays a relevant role in the functional decline of the brain with age.

## Supporting Information

S1 Fig mRNA expression levels of *Drosophila* homologue genes related to the carnitine biosynthesis pathway from aged flies (5weeks) versus young flies (1week). No other potential *Drosophila* homologues of the hGBBD, related to the carnitine biosynthesis pathway, are upregulated with age, they are in all cases 10–30% decreased (*p<0.05, **p<0.01; SEM is indicated with n = 3; Unpaired Student’s *t*-test).
(TIF)

S2 Fig Level of transcriptional knockdown of CG10184 in neuronal tissue resorting to fly line nSyb-Gal4>UAS-CG10814^RNAi^, (NIG, Kyoto). mRNA expression levels of CG10814 are shown extracted from dissected brains and thoraxic ganglionic masses. CG10814 mRNA levels reduced by 70%, when compared to control line (****p<0.0001; SEM is indicated with n = 8; Unpaired Student’s *t*-test).
(TIF)

S3 Fig Negative geotaxis performance of neuronal specific knockdown of CG10814 (*nSyb-GAL4*>UAS-TRiP CG10814^RNAi^) at 1, 3, 5, 7 weeks of age compared to age-matched control flies (***p<0.001; SEM is indicated with n = 240 flies; Paired Student’s *t*-test). CG10814 knockdown in the nervous tissue using this line results in a rescue of age-dependent loss of performance in NGT, namely at 5 and 7 weeks.
(TIF)

S4 Fig Assessment of dietary restriction effects in flies expressing neuronal specific knockdown of CG10814 versus age-matched controls at 1 week of age. Graph represents quantification of food intake through measurement of blue dye uptake, by analysing Absorbance values (at 625nm) from collected biological samples. (n.s. non significant; SEM is indicated with n = 3; Paired Student’s *t*-test).
(TIF)

S5 Fig **Comparison of circadian/activity parameters of neuronal specific knockdown of CG10814 (nSyb-*GAL4*>UAS-10814**^**RNAi**^**) flies with age-matched controls**, at 1 weeks of age—(**A**), Day Sleep bout number; (**B**), Day activity counts (per 12h period); (**C**), Total activity count (per 24h); (**D**), Night Sleep bout number (*p<0.05; n.s.—non significant; SEM is indicated with n = 192 flies; Paired Student’s *t*-test).
(TIF)

S6 Fig CG10814 knockdown in the nervous tissue results in a rescue of age-dependent ubiquitination profile: Full representation of Ubiquitin profile/levels corresponding to Triton X-100 insoluble fraction extracted from heads of neuronal specific knockdown of CG10814 (*nSybGAL4*>UAS-CG10814^RNAi^) flies compared to age-matched controls, at 5 weeks of age (using Anti-Histone H3, as loading control) (TIF)

S7 Fig **CG10814 knockdown in the nervous tissue reduces lipid related oxidative stress:** (A), 4-HNE-protein adduct levels (indicative of accumulation of lipid oxidation products) derived protein fraction extracted from heads of neuronal specific knockdown of CG10814 (*nSybGAL4*>UAS-CG10814^RNAi^) flies compared to age-matched controls, at 5 weeks of age (B), Quantification of 4-HNE-protein adduct levels normalized to Histone H3 levels (*p<0.05; SEM is indicated with n = 8; Paired Student’s *t*-test).
(TIF)

S8 Fig **Comparison of circadian/activity parameters of neuronal specific knockdown of *dHNF4* (nSyb-*GAL4*>UAS-TRiP dHNF4**^**RNAi**^**) flies with age-matched controls**, at 5 weeks of age—(**A**), Day Sleep bout number; (**B**), Day activity counts (per 12h period); (**C**), Night Sleep bout Average duration (min); (**D**), Night Sleep bout number (*p<0.05; SEM is indicated with n = 192 flies; Paired Student’s *t*-test).
(TIF)

S1 Table Primary analysis of NGT performance of a wide-screen of 260 transgenic RNAi lines targeting downregulation of genes reported to have increased genetic expression with age in *Drosophil*a. *In vivo* fly RNAi screen of approximately 260 transgenic RNAi lines resorting to neuronal specific driver *nSyb-GAL4*. Age-matched control flies and RNAi expressing flies were tested at 5W of age and their performance scored and compared to control performance.
(XLS)
