# Supplementary figures and images for: Genes Related to Fatty Acid β-Oxidation Play a Role in the Functional Decline of the Drosophila Brain with Age
Source: PLoS One. 2016 Aug 12;11(8):e0161143. doi: 10.1371/journal.pone.0161143 (PMC4982618; doi:10.1371/journal.pone.0161143)

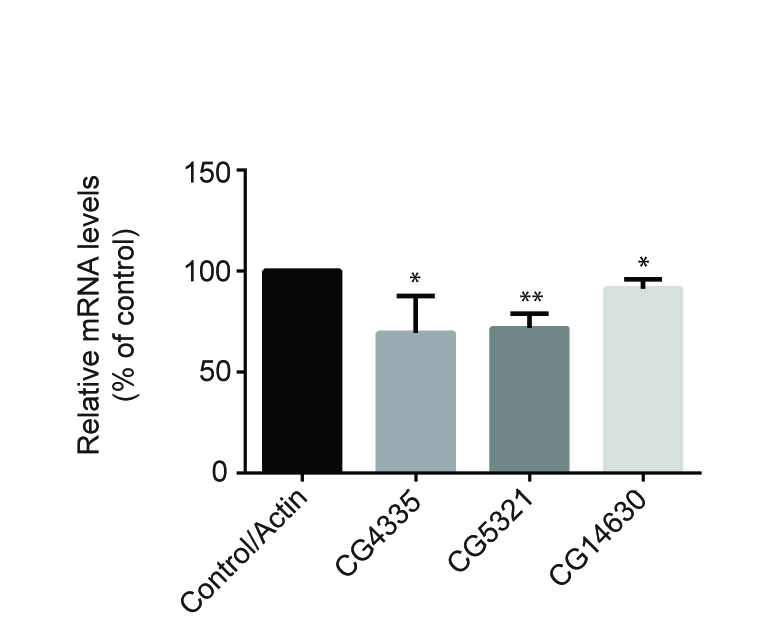

Supplement: S1 Fig — No other potential Drosophila homologues of the hGBBD, related to the carnitine biosynthesis pathway, are upregulated with age, they are in all cases 10–30% decreased (*p<0.05, **p<0.01; SEM is indicated with n = 3; Unpaired Student’s t-test). (TIF) [file pone.0161143.s001.tif]

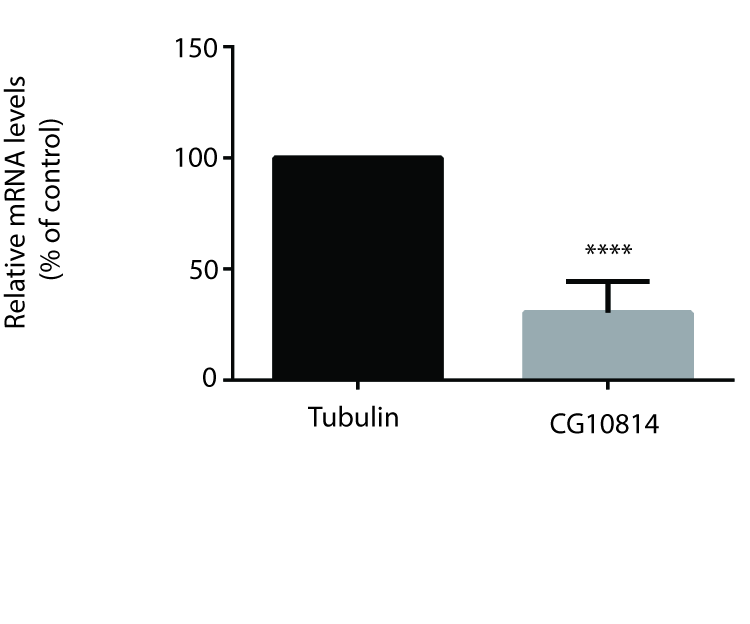

Supplement: S2 Fig — mRNA expression levels of CG10814 are shown extracted from dissected brains and thoraxic ganglionic masses. CG10814 mRNA levels reduced by 70%, when compared to control line (****p<0.0001; SEM is indicated with n = 8; Unpaired Student’s t-test). (TIF) [file pone.0161143.s002.tif]

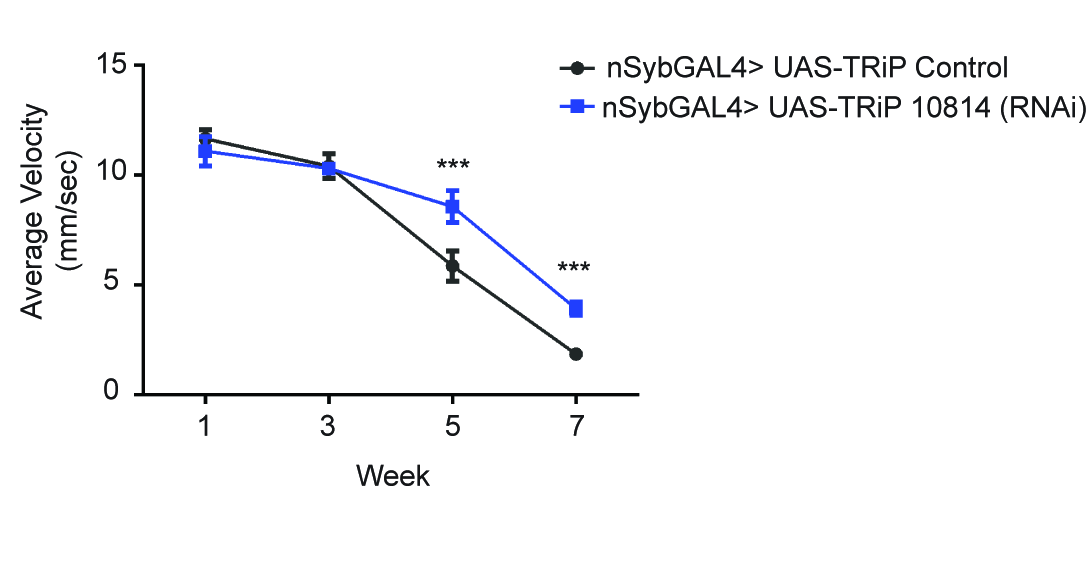

Supplement: S3 Fig — CG10814 knockdown in the nervous tissue using this line results in a rescue of age-dependent loss of performance in NGT, namely at 5 and 7 weeks. (TIF) [file pone.0161143.s003.tif]

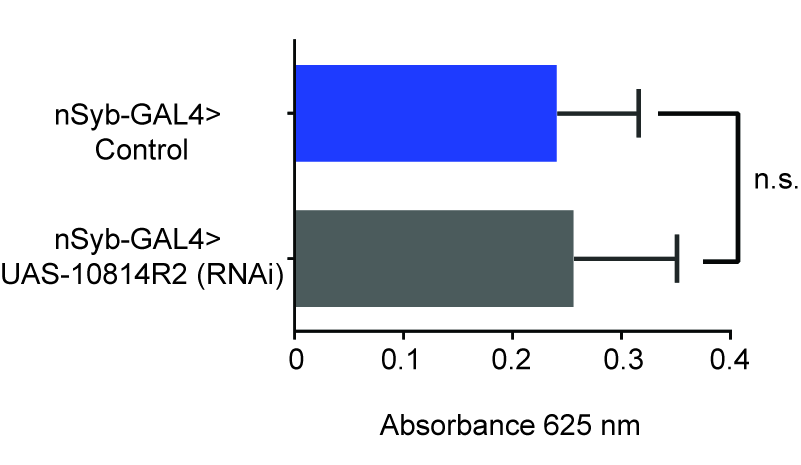

Supplement: S4 Fig — Graph represents quantification of food intake through measurement of blue dye uptake, by analysing Absorbance values (at 625nm) from collected biological samples. (n.s. non significant; SEM is indicated with n = 3; Paired Student’s t-test). (TIF) [file pone.0161143.s004.tif]

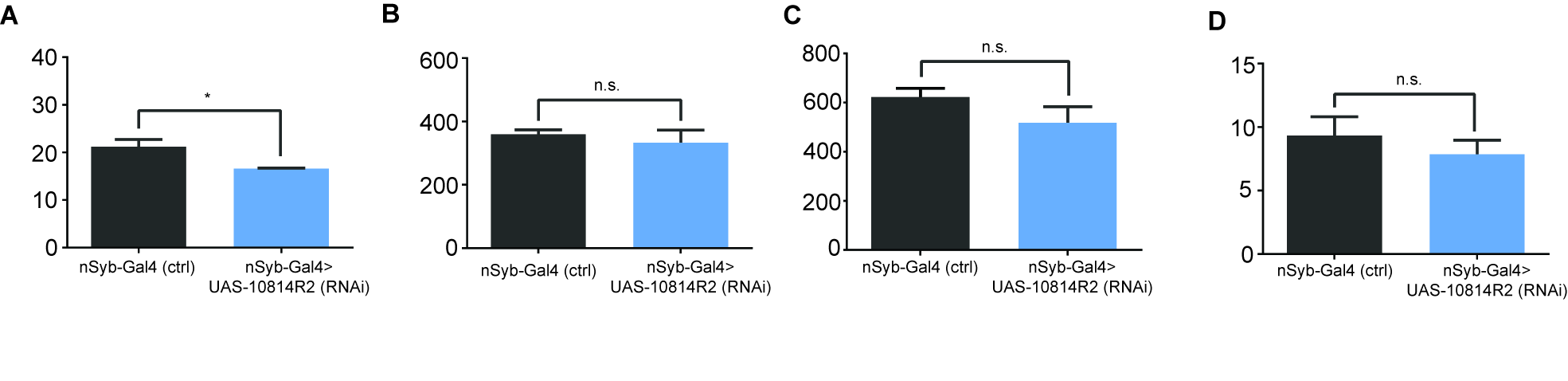

Supplement: S5 Fig — Comparison of circadian/activity parameters of neuronal specific knockdown of CG10814 (nSyb-GAL4>UAS-10814RNAi) flies with age-matched controls, at 1 weeks of age—(A), Day Sleep bout number; (B), Day activity counts (per 12h period); (C), Total activity count (per 24h); (D), Night Sleep bout number (*p<0.05; n.s.—non significant; SEM is indicated with n = 192 flies; Paired Student’s t-test). (TIF) [file pone.0161143.s005.tif]

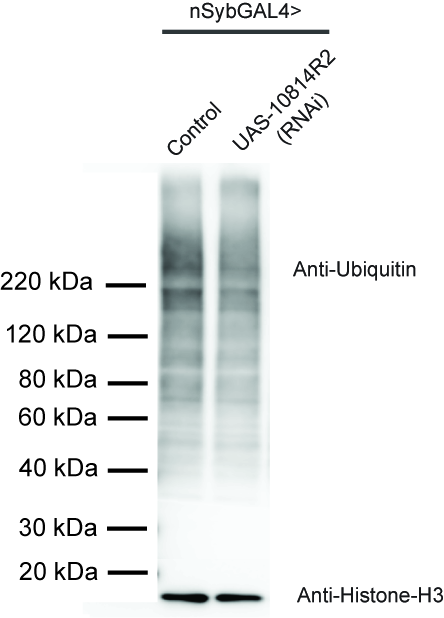

Supplement: S6 Fig — (TIF) [file pone.0161143.s006.tif]

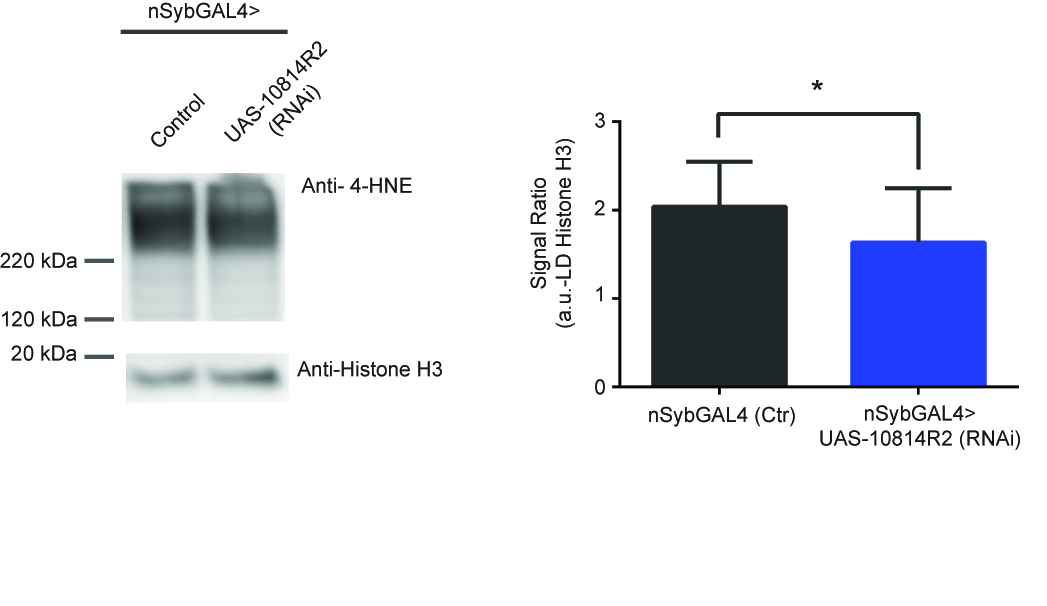

Supplement: S7 Fig — CG10814 knockdown in the nervous tissue reduces lipid related oxidative stress: (A), 4-HNE-protein adduct levels (indicative of accumulation of lipid oxidation products) derived protein fraction extracted from heads of neuronal specific knockdown of CG10814 (nSybGAL4>UAS-CG10814RNAi) flies compared to age-matched controls, at 5 weeks of age (B), Quantification of 4-HNE-protein adduct levels normalized to Histone H3 levels (*p<0.05; SEM is indicated with n = 8; Paired Student’s t-test). (TIF) [file pone.0161143.s007.tif]

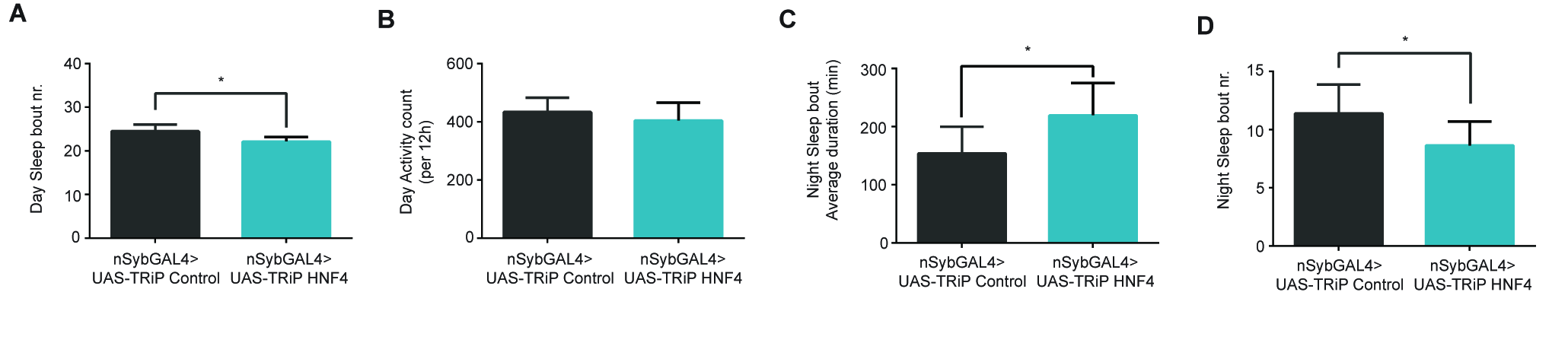

Supplement: S8 Fig — Comparison of circadian/activity parameters of neuronal specific knockdown of dHNF4 (nSyb-GAL4>UAS-TRiP dHNF4RNAi) flies with age-matched controls, at 5 weeks of age—(A), Day Sleep bout number; (B), Day activity counts (per 12h period); (C), Night Sleep bout Average duration (min); (D), Night Sleep bout number (*p<0.05; SEM is indicated with n = 192 flies; Paired Student’s t-test). (TIF) [file pone.0161143.s008.tif]
